# The Genuine Carbene Conundrum

**DOI:** 10.1002/chem.202501600

**Published:** 2025-06-25

**Authors:** Bethany Sawyer, Jesse. L. Peltier, Rodolphe Jazzar

**Affiliations:** ^1^ Department of Chemistry and Biochemistry San Diego State University 5500 Campanile Drive San Diego CA 92182 USA; ^2^ Departments of Chemistry & Chemical Biology and Chemical Engineering Northeastern University 360 Huntington Ave Boston MA 02115 USA; ^3^ UCSD‐CNRS Joint Research Laboratory (IRL 3555) University of California, San Diego La Jolla CA 92093 USA

**Keywords:** carbene trapping reagents, carbenoid, genuine carbene, NHC

## Abstract

Carbenes, these neutral divalent carbon species have emerged as pivotal players in our laboratories. Despite their broad impact, direct characterization of these reactive molecules remains challenging in many cases. This concept article draws on recent literature to explore the strengths and limitations of the most widely used strategies for their identification.

## Graphical abstract

From fleeting intermediates to well‐defined molecular structures, carbenes (neutral divalent carbon) remind us that the most reactive ideas often make a large impact. Despite their wide‐ranging applications, the direct characterization of these reactive species remains a persistent experimental challenge. This concept article explores widely adopted strategies for their detection and structural elucidation, highlighting both their capabilities and limitations.

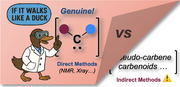

## 1 Introduction

**Figure 1:**
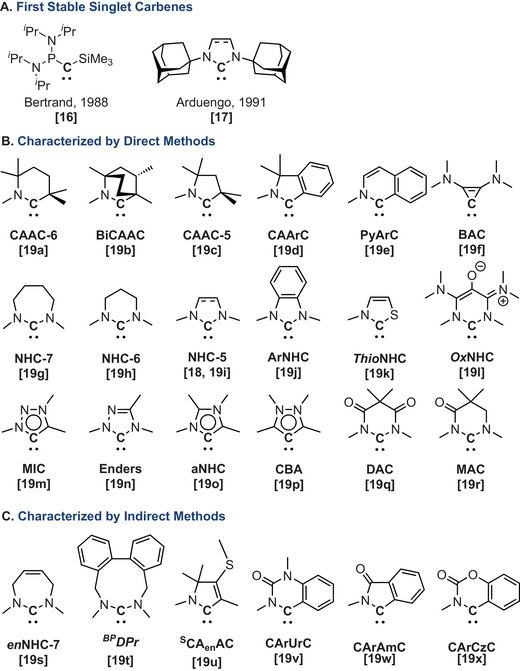
Selected examples of genuine carbenes including the first stable variants a), and those characterized by direct b) and indirect methods c).

The term “carbene” was first introduced at the 119^th^ meeting of the American Chemical Society in 1951 conjointly by Doering, Winstein, and Woodward.^[^ ^1^, ^2^ ^]^ It refers to compounds featuring a neutral, divalent carbon atom (formal oxidation state II) with only six valence electrons which may be in the singlet state or triplet state.^[^ ^3^, ^4^ ^]^ Since their conceptual origins, carbenes have undergone a remarkable evolution, transforming from theoretical curiosities into powerful tools for the chemist practitioner.^[^ ^5^ ^]^ Best known for their roles as ligands in transition metal^[^ ^6^ ^]^ and main‐group chemistry,^[^ ^7^ ^]^ they also earned notoriety in organocatalysis.^[^ ^8^ ^]^ Thanks to their ability to form strong and tunable bonds across the periodic table,^[^ ^9^, ^10^ ^]^ their influence is expanding orthogonally to include (but not limited to) material science,^[^ ^11^ ^]^ molecular electronics,^[^ ^12^ ^]^ supramolecular chemistry,^[^ ^13^ ^]^ and biosynthesis.^[^ ^14^ ^]^ Perhaps influential to their growth, has been the advent of advanced spectroscopic techniques which continue to reveal carbenes in unexpected places. Notably, in astrochemistry, where dicarbenes (i.e., C :C═C═C:) and heterocarbenes (i.e., C :C═S,C :C═O,C :C═NH) in carbon‐rich stellar atmospheres offer clues about molecular evolution in extreme environments.^[^ ^15^ ^]^ More instrumental were the reports of the first bottleable singlet carbenes, which marked a paradigm shift by demonstrating that rationally designed carbenes could be both stable and isolable (Figure 1A).^[^ ^16^, ^17^ ^]^ Until then, carbenes were predominantly studied as reactive intermediates, generated in situ and inferred through their reactivities (*Indirect Methods*).^[^ ^18^ ^]^ However, the introduction of structurally robust carbenes, facilitated their characterization by popular spectroscopic techniques (*Direct methods*).^[^ ^16^, ^17^ ^]^ Today, the field includes many classes of stable carbenes (Figure 1B),^[^ ^19a‐r^ ^]^ such as the well‐known N‐heterocyclic carbenes (NHCs)^[^ ^6^, ^20^ ^]^ or the increasingly popular cyclic(alkyl)(amino) carbenes (CAACs)^[^ ^21^ ^]^ which are more nucleophilic and more electrophilic. It also encompasses several classes of transient carbenes that continue to evade direct characterization, yet are inferred solely from their reactivities (Figure 1C).^[^ ^19s‐x^ ^]^ Using recent examples from the literature, we examine the strengths and limitations of current strategies used to identify and study these transient carbenes. A critical assessment when considering their implications in fields where their presence is yet to be confirmed.

## 2 Discussion

### 2.1 Genuine Carbene: Direct versus Indirect Methods

**Scheme 1:**
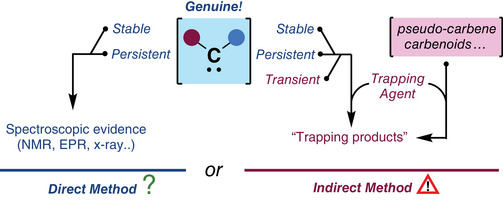
Complementary roles of direct and indirect methods in carbene and carbenoid studies.

Although alternative definitions have been detailed in the literature,^[^ ^19m,o,p^ ^]^ the most traditional description of a “genuine” carbene is that of a free, unbound species with a sextet of electrons in its valence shell with two nonbonded electrons. When sufficiently stable their formation is readily confirmed by *Direct methods* (Scheme 1). In this situation, spectroscopic techniques (e.g., NMR^[^ ^22^ ^]^; X‐ray crystallography^[^ ^23^ ^]^) have become instrumental in elucidating their structure and reactivities.

**Scheme 2:**
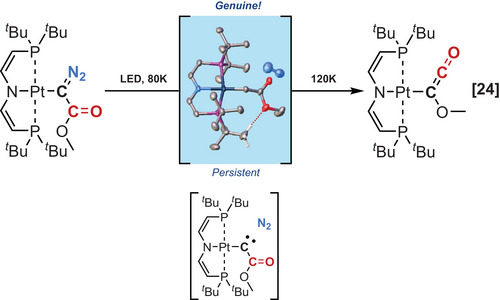
Example of a metallocarbene characterized by direct methods.

An elegant example of this strategy was recently reported by Holthausen, Schneider, and colleagues for the isolation of a metallocarbene (M─C: ─CO_2_Me) intermediate in a matrix during the crystal‐to‐crystal photofragmentation of a metalated diazoester. This report provides firsthand demonstration of the formation of a transient carbene in the Wolff rearrangement (Scheme 2).^[^ ^24^ ^]^ When highly reactive carbenes elude conventional characterization methods, more sensitive spectroscopic techniques are employed.^[^ ^25^, ^26^ ^]^ A remarkable example of this strategy was used by Matsuda and colleagues capitalizing on electron spin resonance (ESR) to confirm the formation of triplet carbenes within the confined environment of metal‐organic frameworks (MOFs).^[^ ^27^ ^]^ Critical to their assessment was the direct comparison of the spectrum of the carbene monomer obtained in a frozen matrix with that of the corresponding carbene embedded in the material.

**Scheme 3:**
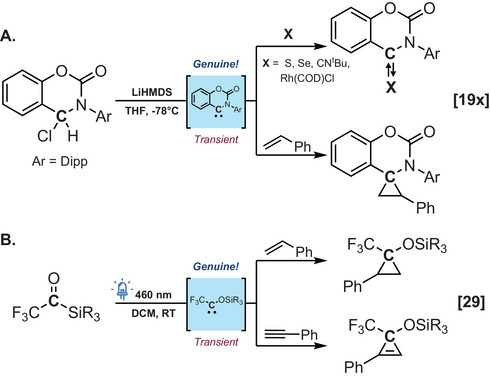
Examples of carbenes [a) coumaraz‐2‐on‐4‐ylidenes, b) siloxycarbenes] characterized by indirect methods.

In the absence of direct observation, chemists can still infer their formation through *Indirect methods*. Among these, trapping experiments are by far the most prevalent approach with numerous studies providing evidence for carbene formation with varying degrees of trapping efficacy. Recent examples of this strategy are Kosumoto's diboryl carbene isolated as a base‐stabilized adduct and trapped by triphenylphosphine,^[^ ^28^ ^]^ or Lee's coumaraz‐2‐on‐4‐ylidene (CArCzC) characterized through its reactivity with small molecules and transition metals (Scheme 3A).^[^ ^19x^ ^]^ Examples of this strategy also include transient synthetic intermediates such as siloxycarbenes which are trapped in situ after being thermally or photochemically generated from acylsilanes via silicon‐to‐oxygen shift, namely a Brook rearrangement (Scheme 3B).^[^ ^29^ ^]^

**Scheme 4:**
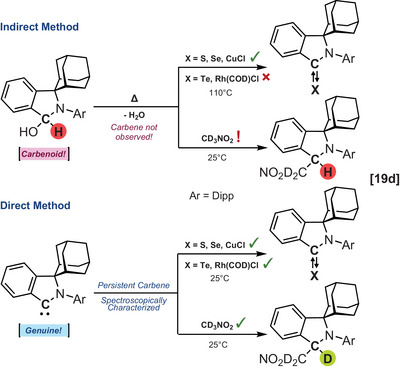
Genuine cyclic(aryl)(amino) carbenes display a broader reaction profile than their carbenoid analogues.

A major limitation of indirect detection methods lies in the difficulty of clearly distinguishing between the reactivity of carbenoids (i.e., reactive intermediates or compound that exhibits carbene‐like reactivity without forming a free carbene as originally defined by Closs and Moss in 1964^[^ ^30^, ^31^ ^]^) and carbenes. While the appeal of carbenoids extends from their enhanced stability under ambient conditions, genuine free carbenes, on the other hand, typically display a broader reaction profile. Cyclic(aryl)(amino) carbenes (CAArCs) present an interesting case to illustrate this point.^[^ ^32^, ^33^ ^]^ The free carbene was recently proposed to form via thermal dehydration of its water adduct and inferred through trapping experiments by using sulfur, selenium, and copper chloride.^[^ ^34^ ^]^ This result contrasted with an earlier report by Radius and co‐workers raising concerns about the accessibility of this carbene.^[^ ^35^ ^]^ To address this conundrum, Bertrand, Jazzar, and colleagues confirmed notable differences in the reactivity profiles of the genuine CAArC (generated through deprotonation of a suitable conjugate acid with a strong base) and that of its water adduct‐derived carbenoid (Scheme 4).^[^ ^19d^ ^]^

This example and others raise concerns about over‐reliance on popular trapping reactions as definitive proof of free carbene formation.^[^ ^36^ ^]^ It also underscores the critical need to distinguish genuine carbene reactivity from that of carbenoids, particularly when relying on indirect methods, as conflating the two can blur mechanistic boundaries and distort our understanding of the field.

### 2.2 Popular Trapping Methods

**Figure 2:**
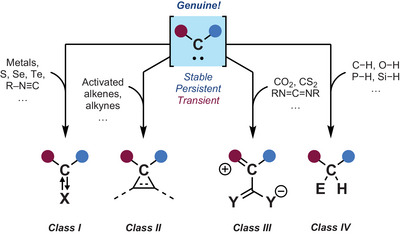
Popular classes of trapping strategies used for inferring carbene formation (indirect methods).

Historically speaking, trapping reactions (*Indirect methods*) have been at the core of discoveries in this field in part due to the absence of suitable or readily accessible spectroscopic techniques. As early as 1912, Staudinger and Kupfer, hinting at the formation of divalent carbon species, reported unusual reactions of diazomethane with alkenes to form cyclopropanes.^[^ ^18^ ^]^ Thanks to these results and many others since, the reactivity of carbenes with small molecules can be classified into four main categories (**Class I‐IV**) (Figure 2).

**Scheme 5:**
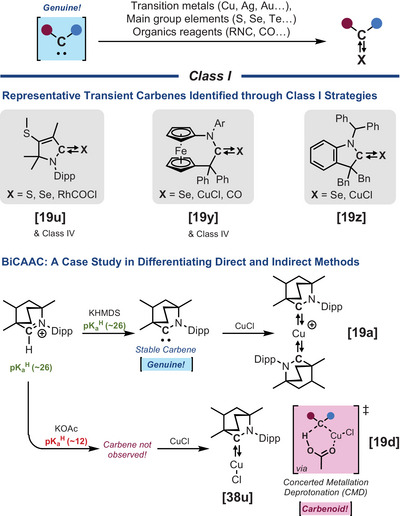
Trapping experiments with transition metals and main group elements (Class I).

**Class I** encompasses the trapping of carbenes with transition metals and main group elements.^[^ ^6^, ^37^ ^]^ These adducts are often depicted using a dative bond from the carbene to the paired element (i.e., R_2_C:→X). However, dual arrows could present an interesting alternative to capture the broad spectrum of electronic properties and bonding interactions accessible between carbenes and their adducts (e.g., R_2_C⇄X, R_2_C⥂X, R_2_C⥄X).^[^ ^38^ ^]^ **Class I** represents both a powerful strategy for stabilizing these reactive intermediates (Scheme 5), and a probe for characterizing their electronic properties.^[^ ^38^ ^]^ In several cases, however, particularly those involving chalcogens or coinage metals. **Class I** adducts derived from carbenoid precursors can form through concerted or associative mechanisms, bypassing the free carbene pathway altogether. While this approach remains a valuable tool for exploring carbene reactivity, it does not necessarily provide conclusive evidence for the formation of a discrete free carbene intermediate. An example of this scenario can be seen with bicyclic (alkyl)(amino)carbenes (BiCAACs), predicted pKa^H^ ≃ 26).^[^ ^19^, ^39^ ^]^ When metalation with copper chloride was performed with the free BiCAAC, obtained by deprotonation of its conjugate acid with a strong base (i.e., KHMDS), a bis‐ligated copper complex was obtained.^[^ ^19b^ ^]^ In contrast, under thermodynamically unfavorable deprotonation conditions (i.e., KOAc) where the free carbene should not be accessible, only a mono‐ligated complex was formed,^[^ ^40^ ^]^ pointing to a concerted metalation‐deprotonation‐type mechanism.^[^ ^19d^ ^]^

**Scheme 6:**
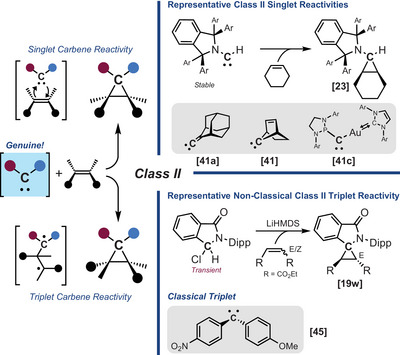
Trapping experiments with alkenes and alkynes (class II).

**Class II** embraces the reactivity of carbenes with unsaturated species such as alkenes and alkynes, notably leading to cyclopropanes and cyclopropenes, respectively. With alkenes, this reactivity also serves as a diagnostic tool for probing their ground‐state multiplicity (Scheme 6).^[^ ^3^, ^41^ ^]^ In this context, singlet carbenes typically undergo stereoselective, concerted cycloaddition reactions consistent with the cheletropic mechanism proposed by Skell.^[^ ^42^ ^]^ In some cases, nucleophilic carbenes have also been shown to undergo 1,2‐hydride shifts when reacting with Michael acceptors.^[^ ^19^, ^43^ ^]^ Interestingly, the electronic nature (or *philicity*) of the carbene has been shown to influence its cycloaddition rates and selectivity toward electron‐rich versus electron‐poor alkenes, with ambiphilic carbenes reacting efficiently with both.^[^ ^44^ ^]^ In contrast, triplet carbenes react with a broader range of alkenes and alkynes through stepwise radical pathways.^[^ ^45^ ^]^ However, complications can arise in this distinction. For example, triplet carbenes with a ground state nearly degenerate to the singlet state (ΔE_ST_ ∼ 3 kcal/mol) may exhibit singlet‐like reactivity.^[^ ^44^ ^]^ Similarly, singlet carbenes such a cyclic (aryl)(amido)carbenes (CArAmCs) or diamidocarbenes (DACs) have been shown to exhibit triplet‐like reactivity.^[^ ^19w^ ^]^ All of which potentially confounds mechanistic interpretations suggesting a need to better differentiate these species in the literature.

While the formation of cyclopropanes is often understood as strong evidence for free carbene reactivity, similar products can also arise through carbene‐free pathways under comparable reaction parameters. This is exemplified by diazomethanes, which can serve as precursors to transient free carbenes prone to react with alkenes to form cyclopropanes.^[^ ^46^ ^]^ However, they can also yield cyclopropanes via 1,3‐dipolar cycloaddition (Huisgen reaction) with alkenes followed by nitrogen extrusion thereby mimicking free carbene reactivity.^[^ ^47^ ^]^ In comparison, the Simmons‐Smith reaction, wherein an organozinc intermediate (IZnCH_2_I) reacts with alkenes to afford cyclopropanes, represents a distinct mode of carbenoid reactivity that also bypasses the formation of free carbenes.^[^ ^31^ ^]^

**Scheme 7:**
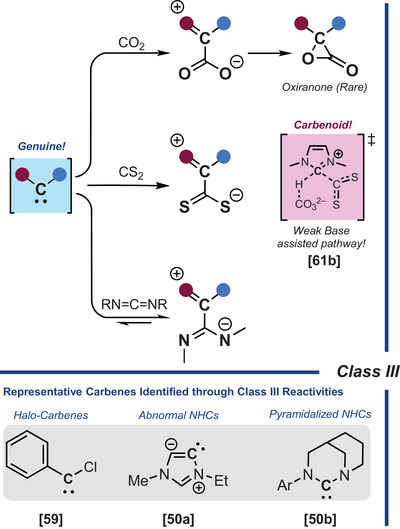
Trapping experiments with CO_2_, CS_2_, and carbodiimides (class III).

**Class III**, first described by Kuhn and colleagues with NHCs,^[^ ^48^ ^]^ involves the formation of zwitterionic betaines which rely on charge transfer from the carbene lone pair to the electrophilic carbon center (Scheme 7).^[^ ^49^ ^]^ Carbon dioxide (CO_2_) and disulfide (CS_2_) are by far the most popular trapping agents for this class,^[^ ^50^, ^51^ ^]^ but carbodiimides,^[^ ^52^ ^]^ isocyanate^[^ ^53^ ^]^ and isothiocyanate^[^ ^54^ ^]^ have also been used.^[^ ^55^ ^]^ For betaine forming adducts, and especially in the case of isocyanates, these species can form heterocycles (e.g., cyclotrimerization) upon complexation to the strongly nucleophilic carbene.^[^ ^56^ ^]^ With CO₂, the strength of the interaction is strongly influenced by the carbene nucleophilicity and can be reversible.^[^ ^57^ ^]^ A feature that has been used by Glorius and others to generate free carbenes under base‐free conditions.^[^ ^58^ ^]^ Albeit very rare, the formation of oxiranone from CO_2_, as well as the direct reduction of CO_2_ to CO has been observed.^[^ ^59^ ^]^ In contrast to CO_2_, CS₂ typically yields more stable, crystalline betaines,^[^ ^50^ ^]^ which can also be obtained by a carbene‐free pathway.^[^ ^60^ ^]^ Notably, CS₂ has been shown to cleave tetraaminoethylenes (i.e., a dimer of NHCs) or react with azolium salts under weak base conditions to afford diaminocarbenium‐dithiocarboxylates without involving a free‐carbene intermediate.^[^ ^61^ ^]^ While not as developed, there is growing interest in the study of carbodiimide betaine adducts’ for their significance as organocatalyst precursors.^[^ ^62^ ^]^ Interestingly, the scope of this reactivity still remains unclear, as some carbenes such as CAACs fail to react with carbodiimides.^[^ ^63^ ^]^

**Scheme 8:**
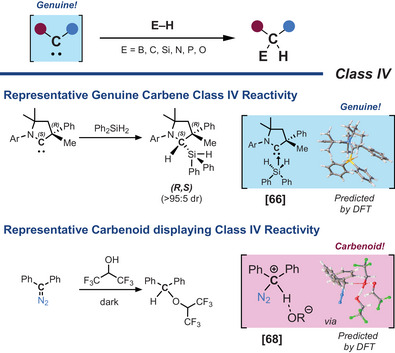
Trapping experiments with E─H bonds (E = B, C, Si, N, P, O) (class IV).

**Class IV** has emerged as a valuable tool for probing highly reactive carbenes by studying their propensity to oxidatively add to E–H bonds.^[^ ^64^ ^]^ In these reactions, the carbene carbon undergoes quaternization through E─H bond insertion (E = C, O, N, Si, P, B), effectively “freezing” the carbene reactivity in a structurally diagnostic product (Scheme 8). Owing to the reversibility of this process (E = O, N, P, B),^[^ ^65^ ^]^ C─H bonds are generally favored as trapping reagents, particularly phenylacetylene (C(sp)─H), pentafluorobenzene (C(sp^2^)─H), or nitromethane (C(sp^3^)─H).^[^ ^19^, ^63^ ^]^ Si─H bonds, as in diphenyl silane, may present an compelling case for further exploration. They are proposed to proceed through a transient pentacoordinate carbene‐silane intermediate (R_2_C⇄SiPh_2_H_2_) prior to Si─H insertion, potentially offering strong evidence for free carbene formation.^[^ ^66^ ^]^ Interestingly, homoleptic bond cleavage (E─E bonds) is another trapping avenue for further exploration.^[^ ^67^ ^]^ **Class IV** reactivity is particularly facile, for highly ambiphilic carbenes which balance strong nucleophilicity and electrophilicity.^[^ ^63^, ^65^ ^]^ However, caution must again be exercised in interpreting these results, as such products may also arise from nucleophilic attack on a transient cationic intermediate bypassing the free carbene formation entirely.^[^ ^68^ ^]^

## 3 Summary and Outlook

Carbon lies at the heart of organic chemistry and carbenes stand as a striking expression of that versatility, revealing a rich spectrum of stereoelectronic properties and reactivities spanning disciplines. As expected, more than 25 years after the first isolation of the first stable carbene, the field continues to thrive, propelled by new discoveries and conceptual shifts. In this context, so too must the questions we ask, notably in distinguishing between the reactivities of free carbenes and their carbenoid analogues. While the outcome may sometimes appear similar, the mechanistic origins often diverge significantly, influencing reactivity, selectivity, and behavior. This mechanistic nuance is not merely academic, as it informs how we design reactions, and imagine new possibilities. Thankfully, the growing interplay between computation and experiment has already enriched this dialogue. Quantum mechanical methods now routinely support spectroscopic data, refining our understanding of carbenes electronic structures, spin states, and reactivity profiles. Combined with the development of more selective and mechanistically informative experimental **Class I‐IV** probes, it will certainly enable more rigorous identification of these species in a broad range of chemical environments. As we continue to sharpen these tools and broaden our perspectives, we may find that some of the most exciting carbenes are still waiting to be discovered particularly in domains where their occurrence is unexpected or obscured by complex reactivity.

## Conflict of Interest

The authors declare no conflict of interest.

## Data Availability

Data sharing is not applicable to this article as no new data were created or analyzed in this study.
